# Binder Jetting 3D Printing of Biomass–Fungi Composite Materials: A Preliminary Experimental Study

**DOI:** 10.3390/biomimetics10070441

**Published:** 2025-07-04

**Authors:** Yeasir Mohammad Akib, Caleb Oliver Bedsole, Jackson Sanders, Harlie Warren, Zhijian Pei, Brian D. Shaw

**Affiliations:** 1 Department of Industrial & Systems Engineering, Texas A&M University, College Station, TX 77843, USA; jacksonjsanders@tamu.edu (J.S.); zjpei@tamu.edu (Z.P.); 2 Department of Plant Pathology and Microbiology, Texas A&M University, College Station, TX 77845, USA; olib@tamu.edu (C.O.B.); harlie.warren@tamu.edu (H.W.); bdshaw@tamu.edu (B.D.S.)

**Keywords:** binder jetting, binder, biomass-fungi, mycelium

## Abstract

This paper reports on a preliminary experimental study on binder jetting 3D printing of biomass–fungi composite materials. Biomass–fungi composite materials have potential applications in the packaging, furniture, and construction industries. Biomass particles (prepared from agricultural residues) act as the substrate of the composite materials. The filamentous roots of fungi intertwine and bind biomass particles together. In this study, the biomass (hemp hurd) powders used had two distinct average particle sizes. The liquid binder used contained fungi (*Trametes versicolor*) cells. T-shaped samples were printed using a lab-designed binder jetting setup. Printed samples were kept inside an incubator oven for four days to allow fungi to grow. Afterward, loose biomass powder was removed from the T-shaped samples. The samples were then kept inside the incubator oven for eight more days to allow further fungal growth. The samples were subsequently placed in an oven at 120 °C for four hours to terminate all fungal activity in the samples. SEM micrographs were taken of the cross-sectional surfaces of the samples. The micrographs showed a significant presence of fungi hyphae inside the printed samples, providing evidence of the binding of biomass particles by the hyphae.

## 1. Introduction

The use of petroleum-derived plastic materials in our daily lives has grown significantly, with a global production of about 400 million tons in 2022 [1]. Most of these plastic materials are discarded after a single use. It was estimated that approximately 6300 million tons of plastic waste had been released into the environment by 2015, and 79% of this waste was accumulated in landfills [2]. If the current trend continues, approximately 12,300 million tons of plastics will be accumulated in landfills by 2050 [2]. Plastics are non-biodegradable (or take years to degrade); therefore, it is essential to find sustainable materials that can biodegrade at the end of their life cycle. 

Biomass–fungi composite materials (that are biodegradable [3,4,5,6,7]) can be used to manufacture some of the products typically produced from petrochemical plastic materials, especially in the furniture, packaging, and construction industries [8,9,10,11,12,13,14,15]. The biomass in biomass–fungi composite materials is primarily sourced from agricultural residues such as wood sawdust, corn stover, hemp hurd, sugar cane, and wheat straw [16,17,18,19,20]. In biomass–fungi composite materials, biomass particles act as the substrate. The thread-like structures (called hyphae) of fungi grow through the biomass particles and bind them together [21,22,23,24,25]. 

Reported studies regarding manufacturing using biomass–fungi composite materials [26,27,28,29] used molding (or pressing)-based manufacturing methods, which are usually costly and have limitations on product geometry. 3D printing can create products of complex shapes, require less setup time and energy, and reduce material waste [30,31,32,33,34,35,36,37]. The first paper on 3D printing of biomass–fungi composite materials was published by the authors in 2020 [38]. Since then, the authors have investigated the effects of mixture composition on extrudability and the rheological characteristics of printable biomass–fungi mixtures [39]; the effects of waiting time (the interval between mixture preparation and 3D printing) on mechanical and rheological properties of biomass–fungi mixtures and print quality [40]; the effects of mixing parameters (such as mixing mode and time) and printing parameters (such as extrusion pressure and printing speed) on fungal growth in printed samples [41]; the effects of ionic crosslinking on print quality (such as height shrinkage and geometric accuracy) [42]; the effects of sodium alginate and calcium chloride on the growth of fungi in biomass–fungi composite materials [43]; and the biodegradability of 3D-printed samples using biomass–fungi composite materials [3].

Other researchers have also reported studies regarding 3D printing-based manufacturing methods using biomass–fungi composite materials. A summary of these studies is shown in Table 1. An agar-based ink (containing agar, coffee grounds, and mycelium) was developed to improve the mechanical and self-healing properties of 3D-printed samples [44]. A robotic 3D printing process was used to study the effects of process parameters (such as nozzle size, printing speed, and extrusion pressure) on printed biomass–fungi composite samples and the effects of autoclaving temperature and waiting time on mixture behavior [19]. An extrudable paste-like mixture was developed using clay, mycelium, hemp, and water for 3D printing [45]. A data-driven study was carried out to measure the influence of geometrical configuration (such as infill density, infill skin thickness, and various patterns) on mycelium growth and the mechanical properties of biomass–fungi composite materials [46]. 

**Figure 1:**
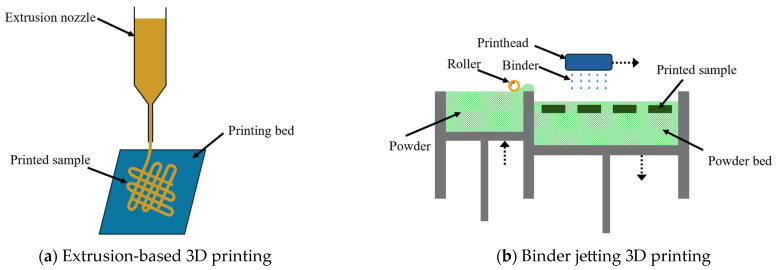
Illustrations of extrusion-based 3D printing and binder jetting 3D printing.

Extrusion-based 3D printing has been used in most reported studies on the 3D printing of biomass–fungi composite materials. In extrusion-based 3D printing, as illustrated in Figure 1a, a paste-like mixture is extruded through a nozzle to print parts layer by layer. A wide range of materials can be used in extrusion-based 3D printing, including wood, timber, fruit, seeds, and herbaceous plants [47]. The major issues with extrusion-based 3D printing using biomass–fungi composite materials include low printing resolution and limited hyphal growth inside extruded filaments [48]. 

This paper reports, for the first time, the use of binder jetting 3D printing with biomass–fungi composite materials. Binder jetting, as illustrated in Figure 1b, prints parts layer by layer by dispensing liquid binder onto selected areas of the powder bed [49]. The advantages of binder jetting include better dimensional accuracy and resolution, and higher production rates [50]. 

**Table 1:** Summary of reported studies on extrusion-based 3D printing of biomass–fungi composite materials.

Reference	Main Contribution
[38]	Preliminary assessment of the suitability of 3D printing for biomass–fungi composite materials
[39]	Effects of biomass–fungi mixture composition on print quality
[40]	Effects of waiting time (the interval between mixture preparation and 3D printing) on mechanical and rheological properties of biomass–fungi mixtures and print quality
[41]	Effects of mixing and printing parameters on fungal growth
[42]	Effects of incorporating ionic crosslinking on the print quality and physicochemical properties of printed samples
[43]	Effects of sodium alginate and calcium chloride on the growth of fungi in biomass–fungi composite materials
[44]	Development of organic waste-based ink (containing mycelium) for printing
[51]	Large-scale robotic 3D printing of mycelium-based materials
[45]	Development of extrudable mixture (containing biomass, clay, and mycelium) for printing
[46]	Effects of geometrical configuration on mycelium growth in printed samples
[47]	Systematic review of a wide range of printable materials from different types of biomass (such as wood, timber, fruit, seeds, and herbaceous plants)

## 2. Materials and Methods

### 2.1. Preparation of Liquid Binder Containing Fungi Cells

**Figure 2:**
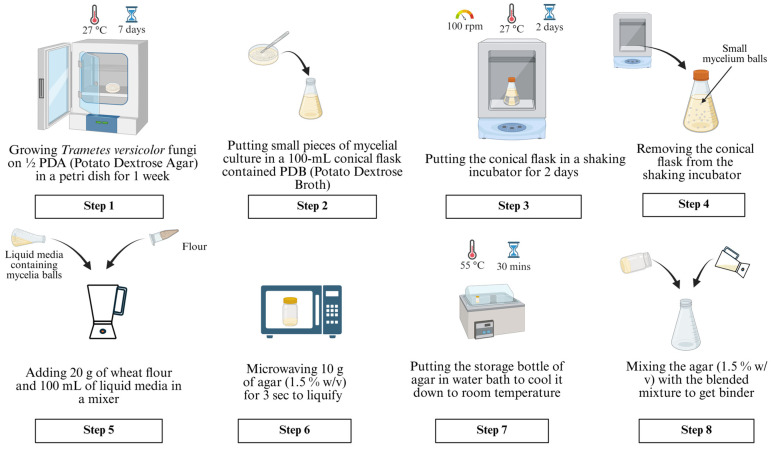
Step-by-step procedure for binder preparation.

The step-by-step procedure for binder preparation is shown in Figure 2 and described below. 

In Step 1, half-strength potato dextrose agar (PDA), prepared with 1000 mL of distilled water, 19.5 g of PDA, and 7.5 g of agar, was used to cultivate Trametes versicolor fungus in a petri dish. The petri dish was kept inside an incubator oven set at 27 °C for one week to obtain the mycelial culture. 

In Step 2, about 50–70 small pieces of 2 cm-sized mycelial culture were cut and placed into a 100 mL conical flask full of PDB (potato dextrose broth) solution. 

In Step 3, the conical flask was placed into a shaking incubator and kept there for two days to facilitate the fungi cell growth. The temperature and speed of the shaking incubator were set at 27 °C and 100 rpm, respectively. These temperature and speed values were selected to ensure that fungal cells would not die. The length of the step (two days) was selected to ensure the formation of small mycelium balls in the conical flask. 

In Step 4, the 100 mL conical flask was removed from the shaking incubator.

In Step 5, 20 g of wheat flour and 100 mL of liquid media containing the mycelium balls were added to a mixing container of a mixer. The mixing time was 3 s—not too long to damage the fungal cells. The mixer did not allow for speed control. The resulting mixture was used in Step 8. 

In Step 6, 10 g of agar (1.5% *w*/*v*) was transferred into a 250 mL storage bottle. Agar is a gelatinous substance that is derived from the cell wall of red algae. The bottle was placed into a countertop microwave and microwaved for 3 sec to obtain liquid agar. 

In Step 7, the storage bottle was placed in a water bath for 30 min while the water temperature was set at 55 °C. 

In Step 8, the agar from the storage bottle and the mixture (prepared in Step 5) were added to a 250 mL conical flask. Mixing was carried out by gently shaking the conical flask by hand to prepare the liquid binder for printing. 

### 2.2. Printing of Samples Using Binder Jetting

**Figure 3:**
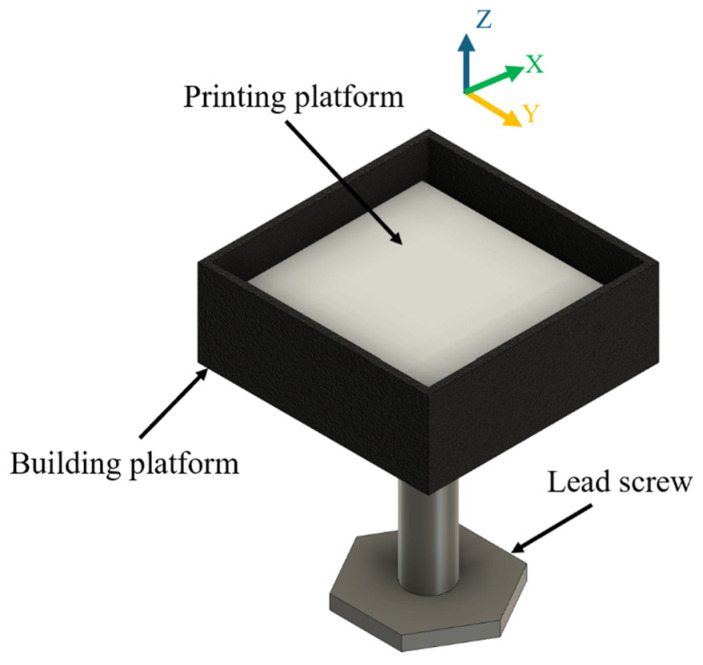
A lab-designed binder jetting setup.

**Figure 4:**
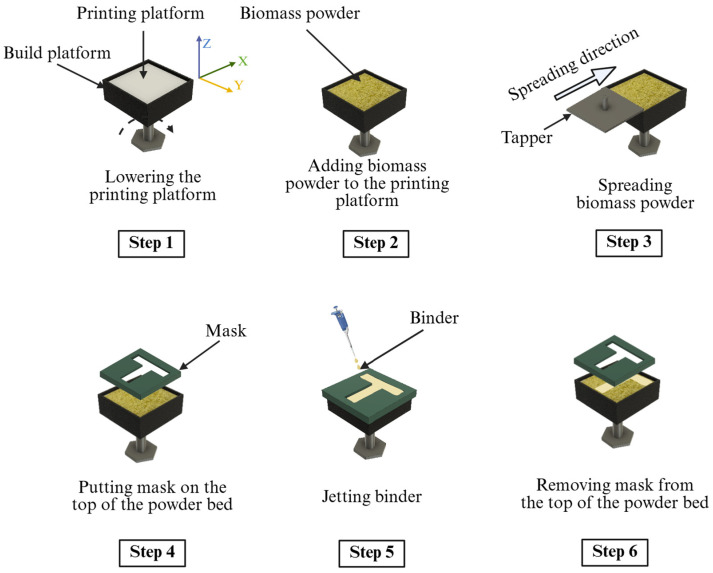
Procedure for printing a sample by binder jetting.

Two groups of biomass powders (hemp hurd particles with average sizes of 0.15 and 2 mm, respectively) were purchased from a commercial supplier (Bulk Hemp Warehouse, Las Vegas, NV, USA) for use in the binder jetting 3D printing. They were used to show the feasibility of the binder jetting approach for biomass powders with different particle sizes. A lab-designed binder jetting setup was used. A schematic illustration of the setup is shown in Figure 3. The volume of the build platform was 75 × 75 × 30 mm^3^. The experiment was performed inside a biological safety cabinet to prevent contamination of the printed samples. The step-by-step procedure for printing a sample is illustrated in Figure 4 and described below. 

In Step 1, the printing platform was lowered by a distance equal to the layer thickness (4 mm in this study). In binder jetting 3D printing, the layer thickness is usually set as two to three times the average powder particle size [50,52,53]. In the experiment, the layer thickness was chosen so that the fungi-embedded binder could penetrate through the powder layer to enable the fungi to grow during the following stages. 

In Step 2, biomass powder was added to the printing platform by hand to ensure that it would cover the entire platform but not overflow.

**Figure 5:**
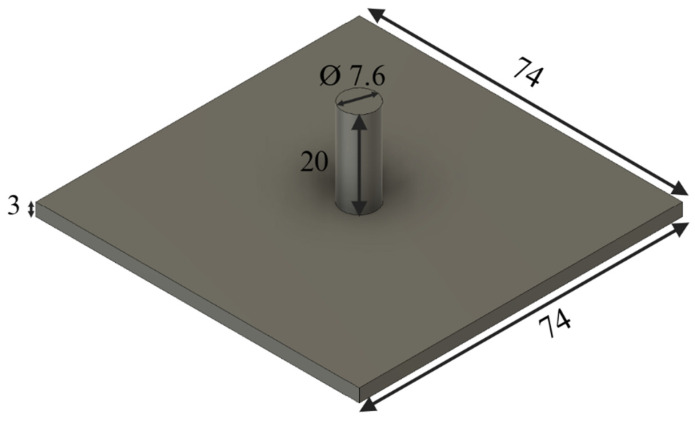
Dimensions (unit: mm) of the tapper used for powder spreading.

In Step 3, a tapper was moved by hand from one side to the other side of the printing platform to spread the biomass powder. A CAD drawing of the tapper with dimensions is shown in Figure 5. All the CAD files used in this study were created using Autodesk Fusion (version 2601.1.34, San Francisco, CA, USA).

**Figure 6:**
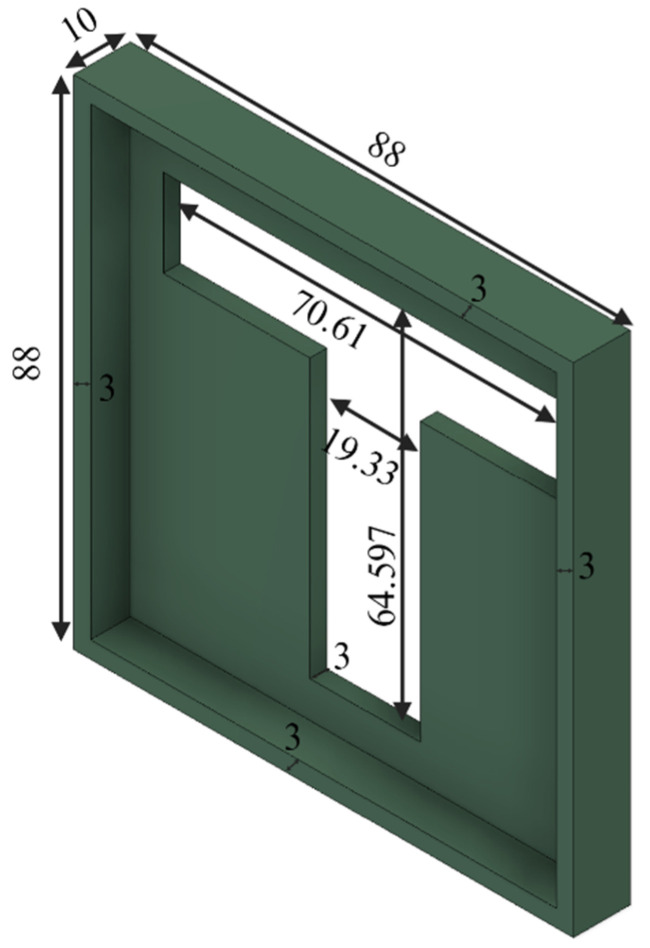
Dimensions of the mask with a T-shaped opening (unit: mm).

In Step 4, the powder bed was covered by a mask with a T-shaped opening. The dimensions of the mask are shown in Figure 6. 

In Step 5, a pipette was used to “jet” the binder (prepared by following the procedure described in Section 2.1) onto the powder bed through the T-shaped opening of the mask. The binder was not jetted on the area of the powder bed unexposed by the mask. The binder was jetted onto the powder bed until the binder covered the entire exposed area of the powder bed. The binder amounts used in the experiment are presented in Table 2. 

In Step 6, the mask was removed from the powder bed.

Steps 1–6 were repeated three more times. The thickness of the printed sample was 16 mm at the end of the printing process.

### 2.3. Processing of Printed Samples to Prepare Final Samples

**Figure 7:**
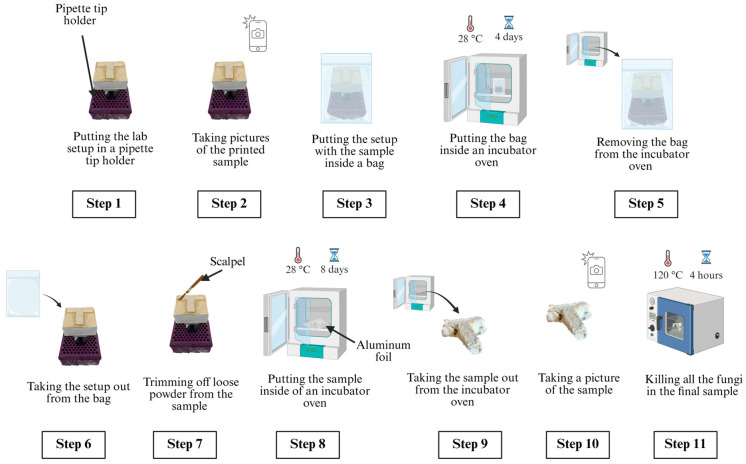
Step-by-step procedure of processing printed samples to prepare final samples.

Figure 7 shows the step-by-step procedure of processing printed samples to prepare the final samples. All these steps were performed outside of the biological safety cabinet. 

In Step 1, the binder jetting setup with the printed sample was placed in a pipette tip holder to keep the setup stable. 

In Step 2, pictures of the printed sample were taken using an iPhone 14 Pro camera (iPhone 14 Pro, Apple, Cupertino, CA, USA).

In Step 3, the setup (with the sample and the pipette tip holder) was placed inside a bag with dimensions of 5″ × 8″ × 20″.

In Step 4, the bag (containing the setup, sample, and pipette tip holder) was placed inside an incubator oven set to 28 °C and kept there for 4 days. The duration of 4 days was selected to ensure that the growth of the fungi was apparent on the T-shaped sample. The duration of the incubation for Step 4 (in Figure 7) and Step 8 (in Figure 7) was selected to allow sufficient growth of fungi based on a prior study by the authors [38]. 

In Step 5, the bag was removed from the incubator oven.

In Step 6, the setup (with the sample and the pipette tip holder) was taken out from the bag. 

In Step 7, loose powder from the T-shaped sample was trimmed off using a sterile scalpel. 

In Step 8, the sample was placed onto aluminum foil, placed in the incubator oven (set at 28 °C) and kept there for 8 days for further fungal growth. This step constitutes the second stage of fungal growth in the method.

In Step 9, the sample was taken out from the incubator oven. 

In Step 10, a picture of the sample was taken.

In Step 11, the sample was placed in a countertop convection oven set at 120 °C for 4 h to kill all the fungi. The T-shaped sample at this point can be referred to as the ‘final sample’.

### 2.4. Taking Micrographs Using Scanning Electron Microscopy (SEM)

**Figure 8:**
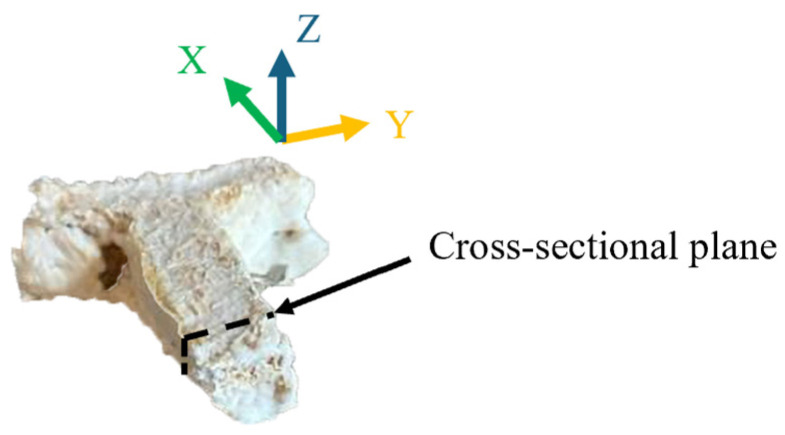
A cross-sectional plane parallel to the Y–Z plane of the final sample.

A cross-sectional micrograph of the final sample printed with 2 mm powder was taken to reveal hyphal growth inside the sample. The micrograph was taken after all the fungi in the secondary colonized sample were killed in the induction oven. To take the cross-sectional micrograph, the final sample was broken along the Y–Z plane using the hand (along the black dotted line shown in Figure 8), where the *Z*-axis is the direction of printing, and the *X*-axis is the direction of the powder spreading. The SEM machine (SNE-4500M Plus, NanoImages, Lafayette, CA, USA) was operated in high-vacuum mode with an accelerating voltage of 15 kV. No sputter coating was used during the sample preparation for SEM. It was recognized that sputter coating is necessary to obtain high-quality images for non-conductive and beam-sensitive samples. Although the SEM image obtained in this study was not of high-quality, it was adequate to show the presence of fungal hyphae. 

## 3. Results and Discussion

**Figure 9:**
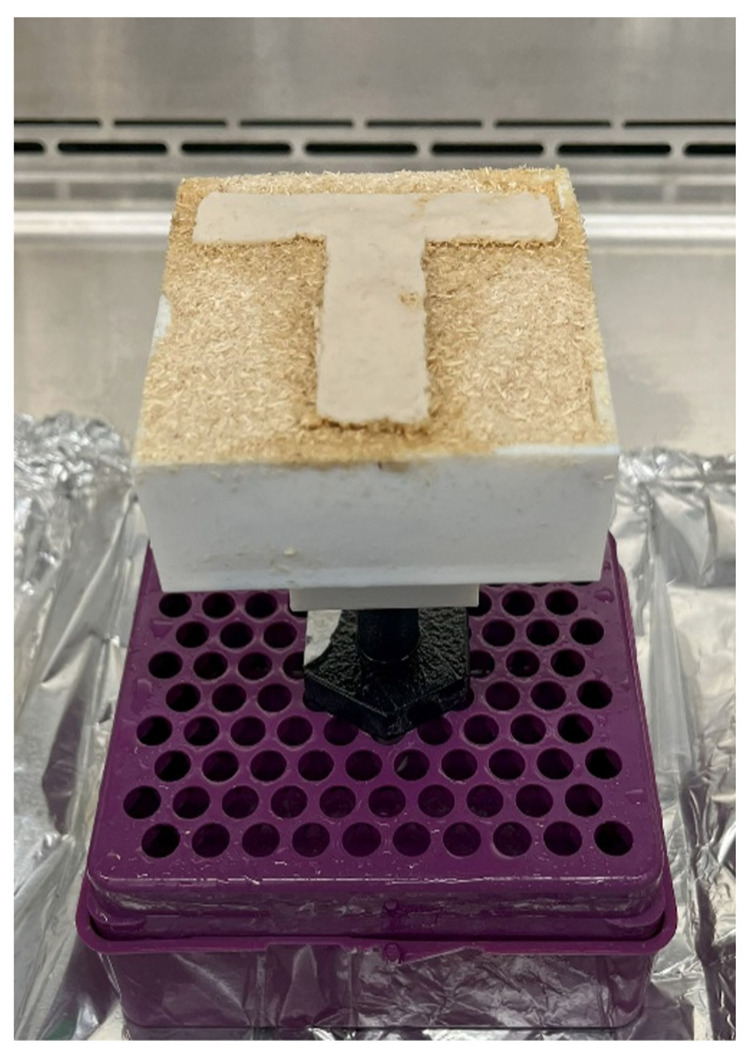
A printed sample generated by binder jetting 3D printing.

**Figure 10:**
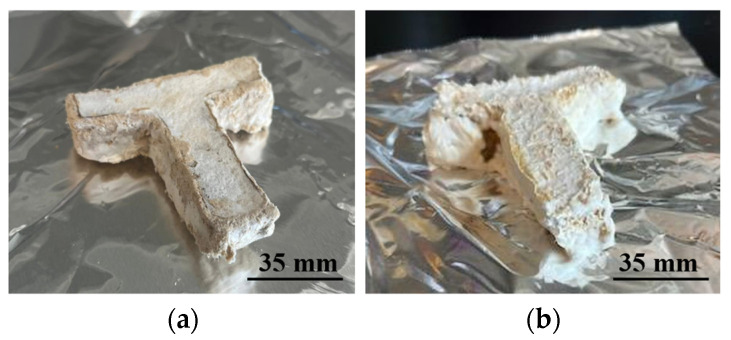
Pictures of final samples printed from (**a**) biomass powder with a particle size of 0.15 mm, and (**b**) biomass powder with a particle size of 2 mm.

Figure 9 shows a picture of a printed sample. Figure 10 shows images of the two final samples printed with biomass powders of different particle sizes. The results show that fungal growth was observed outside of all the samples. 

**Figure 11:**
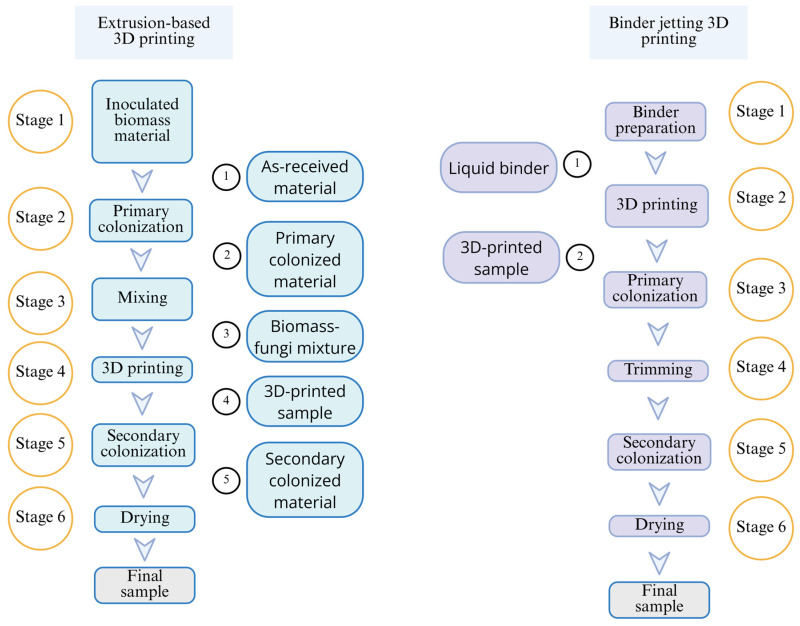
Comparison between extrusion-based and binder jetting 3D printing.

Figure 11 compares the stages of extrusion-based 3D printing versus binder jetting 3D printing of biomass–fungi composite materials conducted at the authors’ lab. For extrusion-based 3D printing, three stages must be completed before 3D printing [38]. The inoculation process of biomass with fungi was completed using Ecovative Design (New York, NY, USA), and the inoculated biomass materials were packed into a filter patch bag. These ‘as-received materials’ need to be supplemented with water and nutrition and kept away from light for 3–5 days at 23 °C during the primary colonization stage. After this primary colonization stage, the primary colonized materials need to be mixed with additional water and psyllium husk powder to obtain the printable biomass–fungi mixture. For binder jetting, only one stage needs to be completed before 3D printing: preparation of a liquid binder containing fungi cells. Like extrusion-based printing, binder jetting of biomass–fungi materials also requires two stages of colonization (primary and secondary colonization) before the drying stage. However, in binder jetting, between the two stages of colonization (primary and secondary colonization), loose powder needs to be trimmed off the printed sample. 

Table 3 compares extrusion-based and binder jetting 3D printing of biomass–fungi composite materials.

**Figure 12:**
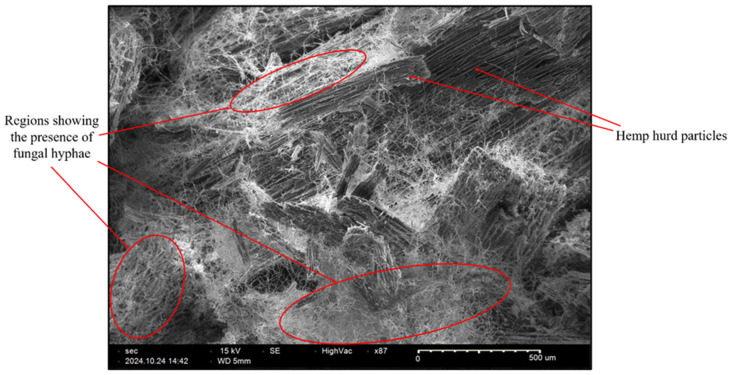
SEM micrograph of the inside of a final sample.

Figure 12 shows an SEM micrograph of a sample printed from the biomass powder with a particle size of 2 mm. Qualitative analysis of the micrograph revealed that there were regions showing the presence of fungal hyphae (marked with oval shapes). Pohl et al. [1] quantified the presence of mycelium (*Fomes fomentarius*) grown in the hemp hurd from SEM micrographs. Their methodology is similar to the one used by the authors of this paper. 

## 4. Conclusions and Future Research Directions

This paper is the first in the literature to report a study on binder jetting 3D printing of biomass–fungi composite materials. The feasibility of this approach was demonstrated by printing samples using hemp hurd powders of two different particle sizes. Evidence of fungal growth was identified on the inside and outside of the printed samples. 

This new approach requires further study to fill many knowledge gaps. There are many future directions regarding biomass materials, liquid binders, binder droplets, preprinting, printing, and post-printing. One research direction is to investigate the effects of different types of biomass and particle size distribution on the dimensional accuracy, fabrication resolution, and mechanical properties of printed samples. Research directions regarding liquid binders include the effects of different binder compositions on the dimensional accuracy and mechanical properties of the printed samples. Another research direction is the effects of binder droplet volume, velocity, spacing, jetting frequency, and stand-off distance on the final printed sample. 

More research directions could include the effects of mixing time, waiting time, and incubation temperature on fungal cell survival and fungal growth during the binder preparation prior to binder jetting 3D printing. An excessive mixing time of the liquid media containing the mycelia balls with the flour in the mixer can damage the fungal cells. The waiting time between the binder preparation and binder jetting 3D printing should be optimal, otherwise the binder can thicken, making it challenging to jet onto the sample. A high incubation temperature can inhibit the growth of fungi [54]. During the printing process, the effects of layer thickness and spreading speed on the mechanical properties of the printed samples should also be investigated. In future studies, the authors plan to investigate the effects of process parameters and particle size on printed samples in great detail. Additionally, different types of biomass and fungi will be investigated regarding their effects on the mechanical, thermal, and chemical properties of printed samples. Finally, future research regarding post-printing processes should include the effects of heating time and temperature on eliminating fungi in the printed samples.

## Figures and Tables

**Table 2:** Binder amounts jetted onto the exposed area of the powder bed for each layer.

Particle Size (mm)	Binder Amount (mL)
0.15	12.75 ± 4.35
2	10.5 ± 1

**Table 3:** Comparison of extrusion-based 3D printing versus binder jetting 3D printing of biomass–fungi composite materials.

	Extrusion-Based 3D Printing	Binder Jetting 3D Printing
Working principle	Material is extruded through a nozzle with force or pressure	Liquid binder is deposited onto selected areas of the powder bed of biomass particles
Material form	Paste-like mixture containing biomass particles and fungi	Biomass powder on powder bed; and fungi embedded binder
Production throughput	Slower process	Faster Process
	Suitable for single-piece or batch production	Suitable for mass production
	Low achievable resolution	High achievable resolution
	Unable to print samples with complex geometry	Able to print samples with complex geometry
Cost per part	Low machine cost	High machine cost
	Easier to scale for large parts	Difficult to print very large parts

## Data Availability

The authors confirm that the analyzed data that support the findings of this study are available within the article and others are available upon request.

## References

[1] Pilapitiya P.N.T., Ratnayake A.S. (2024). The world of plastic waste: A review. Clean. Mater..

[2] Wojnowska-Baryła I., Bernat K., Zaborowska M. (2022). Plastic waste degradation in landfill conditions: The problem with microplastics, and their direct and indirect environmental effects. Int. J. Environ. Res. Public Health.

[3] Akib Y.M., Bedsole C.O., Rahman A.M., Hamilton J., Khan F., Pei Z., Shaw B.D., Ufodike C.O. (2024). A Preliminary Experimental Study on Biodegradation of 3D-Printed Samples from Biomass–Fungi Composite Materials. J. Compos. Sci..

[4] Van Wylick A., Elsacker E., Yap L.L., Peeters E., De Laet L. (2022). Mycelium composites and their biodegradability: An exploration on the disintegration of mycelium-based materials in soil. Constr. Technol. Archit..

[5] Zimele Z., Irbe I., Grinins J., Bikovens O., Verovkins A., Bajare D. (2020). Novel Mycelium-Based Biocomposites (MBB) as Building Materials. J. Renew. Mater..

[6] Ly L., Jitjak W. (2022). Biocomposites from agricultural wastes and mycelia of a local mushroom, Lentinus squarrosulus (Mont.) Singer. Open Agric..

[7] Angelova G., Yemendzhiev H., Zaharieva R., Brazkova M., Koleva R., Stefanova P., Baldzhieva R., Vladev V., Krastanov A. (2025). Mycelium-Based Composites Derived from Lignocellulosic Residual By-Products: An Insight into Their Physico-Mechanical Properties and Biodegradation Profile. Appl. Sci..

[8] Grimm D., Wosten H.A.B. (2018). Mushroom cultivation in the circular economy. Appl. Microbiol. Biotechnol..

[9] Holt G.A., McIntyre G., Flagg D., Bayer E., Wanjura J.D., Pelletier M.G. (2012). Fungal Mycelium and Cotton Plant Materials in the Manufacture of Biodegradable Molded Packaging Material: Evaluation Study of Select Blends of Cotton Byproducts. J. Biobased Mater. Bioenergy.

[10] Heisel F., Hebel D.E. (2019). Pioneering construction materials through prototypological research. Biomimetics.

[11] Abhijith R., Ashok A., Rejeesh C. (2018). Sustainable packaging applications from mycelium to substitute polystyrene: A review. Mater. Today Proc..

[12] Abrams M. (2014). Construction materials made from ‘shrooms’. Am. Soc. Mech. Eng..

[13] Jiang L., Walczyk D., McIntyre G., Bucinell R., Tudryn G. (2017). Manufacturing of biocomposite sandwich structures using mycelium-bound cores and preforms. J. Manuf. Process..

[14] Jiang L., Walczyk D., McIntyre G., Bucinell R. A new approach to manufacturing biocomposite sandwich structures: Mycelium-based cores. Proceedings of the International Manufacturing Science and Engineering Conference.

[15] Jonnala S.N., Gogoi D., Devi S., Kumar M., Kumar C. (2024). A comprehensive study of building materials and bricks for residential construction. Constr. Build. Mater..

[16] U.S. Energy Information. Administration. Biomass Explained. https://www.eia.gov/energyexplained/biomass/.

[17] Jones M.P., Lawrie A.C., Huynh T.T., Morrison P.D., Mautner A., Bismarck A., John S. (2019). Agricultural by-product suitability for the production of chitinous composites and nanofibers utilising *Trametes versicolor* and *Polyporus brumalis* mycelial growth. Process Biochem..

[18] Aiduang W., Chanthaluck A., Kumla J., Jatuwong K., Srinuanpan S., Waroonkun T., Oranratmanee R., Lumyong S., Suwannarach N. (2022). Amazing Fungi for Eco-Friendly Composite Materials: A Comprehensive Review. J. Fungi.

[19] Nagarajan V., Mohanty A.K., Misra M. (2013). Sustainable Green Composites: Value Addition to Agricultural Residues and Perennial Grasses. ACS Sustain. Chem. Eng..

[20] Sydor M., Cofta G., Doczekalska B., Bonenberg A. (2022). Fungi in Mycelium-Based Composites: Usage and Recommendations. Materials.

[21] Verma N., Eswari J.S., Mahapatra C. (2023). Green sustainable biocomposites: Substitute to plastics with innovative fungal mycelium based biomaterial. J. Environ. Chem. Eng..

[22] Schmidt B., Freidank-Pohl C., Zillessen J., Stelzer L., Guitar T.N., Lühr C., Müller H., Zhang F., Hammel J.U., Briesen H. (2023). Mechanical, physical and thermal properties of composite materials produced with the basidiomycete Fomes fomentarius. Fungal Biol. Biotechnol..

[23] Saez D., Grizmann D., Trautz M., Werner A. (2022). Exploring the Binding Capacity of Mycelium and Wood-Based Composites for Use in Construction. Biomimetics.

[24] Wang H., Tao J., Wu Z., Weiland K., Wang Z., Masania K., Wang B. (2024). Fabrication of Living Entangled Network Composites Enabled by Mycelium. Adv. Sci..

[25] Sun W., Tajvidi M., Howell C., Hunt C.G. (2020). Functionality of Surface Mycelium Interfaces in Wood Bonding. ACS Appl. Mater. Interfaces.

[26] Elsacker E., Vandelook S., Van Wylick A., Ruytinx J., De Laet L., Peeters E. (2020). A comprehensive framework for the production of mycelium-based lignocellulosic composites. Sci. Total Environ..

[27] Haneef M., Ceseracciu L., Canale C., Bayer I.S., Heredia-Guerrero J.A., Athanassiou A. (2017). Advanced Materials From Fungal Mycelium: Fabrication and Tuning of Physical Properties. Sci. Rep..

[28] Jones M., Mautner A., Luenco S., Bismarck A., John S. (2020). Engineered mycelium composite construction materials from fungal biorefineries: A critical review. Mater. Des..

[29] Jones M., Huynh T., Dekiwadia C., Daver F., John S. (2017). Mycelium composites: A review of engineering characteristics and growth kinetics. J. Bionanosci..

[30] Attaran M. (2017). The rise of 3-D printing: The advantages of additive manufacturing over traditional manufacturing. Bus. Horiz..

[31] Cheng P., Han Z., Chen Y., Ye L. (2025). Recent progress in non-planar 3D printing of continuous fiber-reinforced composites. Compos. Part. A Appl. Sci. Manuf..

[32] Cheng P., Li S., Peng Y., Duigou A.L., Wang K., Ahzi S. (2023). 3D/4D Printed Functional Continuous Fiber-reinforced Polymer Composites: Progress and Perspectives. Chin. J. Mech. Eng. Addit. Manuf. Front..

[33] Cheng P., Ye Z., Huang Y., Wang D., Peng Y., Wang K., Ahzi S. (2023). Electrical resistance-based self-monitoring of manufacturing damage in 3D printed continuous carbon fiber reinforced composites. Compos. Commun..

[34] Mecheter A., Tarlochan F. (2023). Fused Filament Fabrication Three-Dimensional Printing: Assessing the Influence of Geometric Complexity and Process Parameters on Energy and the Environment. Sustainability.

[35] Fidan I., Naikwadi V., Alkunte S., Mishra R., Tantawi K. (2024). Energy Efficiency in Additive Manufacturing: Condensed Review. Technologies.

[36] Tabassum T., Ahmad Mir A. (2023). A review of 3d printing technology-the future of sustainable construction. Mater. Today Proc..

[37] Muth J., Klunker A., Völlmecke C. (2023). Putting 3D printing to good use—Additive Manufacturing and the Sustainable Development Goals. Front. Sustain..

[38] Bhardwaj A., Vasselli J., Lucht M., Pei Z., Shaw B., Grasley Z., Wei X., Zou N. (2020). 3D Printing of Biomass-Fungi Composite Material: A Preliminary Study. Manuf. Lett..

[39] Bhardwaj A., Rahman A.M., Wei X., Pei Z., Truong D., Lucht M., Zou N. (2021). 3D Printing of Biomass–Fungi Composite Material: Effects of Mixture Composition on Print Quality. J. Manuf. Mater. Process..

[40] Rahman A.M., Bhardwaj A., Pei Z., Ufodike C., Castell-Perez E. (2022). The 3D Printing of Biomass–Fungi Composites: Effects of Waiting Time after Mixture Preparation on Mechanical Properties, Rheological Properties, Minimum Extrusion Pressure, and Print Quality of the Prepared Mixture. J. Compos. Sci..

[41] Rahman A.M., Bhardwaj A., Vasselli J.G., Pei Z., Shaw B.D. (2023). Three-Dimensional Printing of Biomass–Fungi Biocomposite Materials: The Effects of Mixing and Printing Parameters on Fungal Growth. J. Manuf. Mater. Process..

[42] Rahman A.M., Akib Y.M., Bedsole C.O., Pei Z., Shaw B.D., Ufodike C.O., Castell-Perez E. (2024). Effects of Incorporating Ionic Crosslinking on 3D Printing of Biomass–Fungi Composite Materials. Biomimetics.

[43] Rahman A.M., Bedsole C.O., Akib Y.M., Hamilton J., Rahman T.T., Shaw B.D., Pei Z. (2024). Effects of Sodium Alginate and Calcium Chloride on Fungal Growth and Viability in Biomass-Fungi Composite Materials Used for 3D Printing. Biomimetics.

[44] Soh E., Teoh J.H., Leong B., Xing T., Le Ferrand H. (2023). 3D printing of mycelium engineered living materials using a waste-based ink and non-sterile conditions. Mater. Des..

[45] Ibrahim F., Castellano G., Carcassi O.B., Paoletti I.M. (2023). MycoCode: Development of an Extrudable Paste for 3D Printing Mycelium-Bound Composites. Architecture and Design for Industry 4.0: Theory and Practice.

[46] Gomaa H., Chau W.M., Karazi Y., Biala E., Akbar Z., Wortmann T., Ostermann M. (2024). Influence of Geometry on Growth and Strength of 3D-Printed Mycelium Composites: A Data-Driven Study. Design Modelling Symposium Berlin.

[47] Romani A., Suriano R., Levi M. (2023). Biomass waste materials through extrusion-based additive manufacturing: A systematic literature review. J. Clean. Prod..

[48] Pei Z., Rahman A.M., Shaw B.D., Bedsole C.O. (2024). Three-Dimensional Printing Using Biomass–Fungi Composite Materials: Brief Retrospective and Prospective Views. Bioengineering.

[49] Ziaee M., Crane N.B. (2019). Binder jetting: A review of process, materials, and methods. Addit. Manuf..

[50] Mostafaei A., Elliott A.M., Barnes J.E., Li F., Tan W., Cramer C.L., Nandwana P., Chmielus M. (2021). Binder jet 3D printing—Process parameters, materials, properties, modeling, and challenges. Prog. Mater. Sci..

[51] Elsacker E., Peeters E., De Laet L. (2022). Large-scale robotic extrusion-based additive manufacturing with living mycelium materials. Sustain. Futures.

[52] Cheny T., Colin C., Verquin B. (2024). Experimental evaluation of binder infiltration depth and axial overlap to control properties of green parts produced by Binder Jetting. Addit. Manuf..

[53] Zhou Z., Mitchell C.A., Buchanan F.J., Dunne N.J. (2013). Effects of Heat Treatment on the Mechanical and Degradation Properties of 3D-Printed Calcium-Sulphate-Based Scaffolds. Int. Sch. Res. Not..

[54] Šnajdr J., Baldrian P. (2007). Temperature affects the production, activity and stability of ligninolytic enzymes in *Pleurotus ostreatus* and *Trametes versicolor*. Folia Microbiol..

